# Unraveling Variations in Celiac Trunk and Hepatic Artery by CT Angiography to Aid in Surgeries of Upper Abdominal Region

**DOI:** 10.3390/diagnostics11122262

**Published:** 2021-12-03

**Authors:** Kapil Kumar Malviya, Ashish Verma, Amit Kumar Nayak, Anand Mishra, Raghunath Shahaji More

**Affiliations:** 1 Department of Anatomy, Institute of Medical Science, Banaras Hindu University, Varanasi 221005, India; dramit.k.nayak@gmail.com (A.K.N.); dranand5@rediffmail.com (A.M.); psychiatry.more@gmail.com (R.S.M.); 2 Department of Radiodiagnosis and Imaging, Institute of Medical Science, Banaras Hindu University, Varanasi 221005, India; drdnv5@gmail.com

**Keywords:** celiac trunk, hepatic artery, 64-row scanner computed tomography, anatomical variations

## Abstract

Understanding of variations in the course and source of abdominal arteries is crucial for any surgical intervention in the peritoneal space. Intricate surgeries of the upper abdominal region, such as hepato-biliary, pancreatic, gastric and splenic surgeries, require precise knowledge of regular anatomy and different variations related to celiac trunk and hepatic artery. In addition, information about the origin of inferior phrenic artery is important in conditions such as hepatocellular carcinoma and gastroesophageal bleeding management. The present study gives an account of anatomical variations in origin and branching pattern of celiac trunk and hepatic artery by the use of CT (computed tomographic) angiography. The study was performed on 110 (66 females and 44 males) patients in a north Indian population. Results unraveled the most common celiac trunk variation as hepatosplenic trunk with left gastric artery, which was observed in 60% of cases, more common in females than in males. Gastrosplenic and hepato-gastric trunk could be seen in 4.55% and 1.82% cases respectively. Gastrosplenic trunk was more commonly found in females, whereas hepato-gastric trunk was more common in males. A gastrosplenic trunk, along with the hepato-mesenteric trunk, was observed in 1.82% cases and was more common in males. A celiacomesenteric trunk, in which the celiac trunk and superior mesenteric artery originated as a common trunk from the aorta, was seen only in 0.91% of cases, and exhibited an origin of right and left inferior phrenic artery from the left gastric artery. The most common variation of hepatic artery, in which the right hepatic artery was replaced and originated from the superior mesenteric artery, was observed in 3.64%, cases with a more common occurrence in males. In 1.82% cases, the left hepatic artery was replaced and originated from the left gastric artery, which was observed only in females. Common hepatic artery originated from the superior mesenteric artery, as observed in 1.82% cases, with slightly higher occurrence in males. These findings not only add to the existing knowledge apart from giving an overview of variations in north Indian population, but also give an account of their correlation with gender. The present study will prove to be important for various surgeries of the upper abdominal region.

## 1. Introduction

Comprehensive knowledge of regular anatomy and variations in blood vessels is not only important for complicated surgeries, but also aids in the procedures. Variations in celiac trunk and hepatic artery are of utmost importance during hepato-biliary, pancreatic, gastric and splenic surgeries. The celiac trunk supplies blood flow to the stomach, spleen and liver; hence, understanding its variations is crucial to perform complex surgical procedures involving these organs. The celiac trunk originates at the T12 thoracic vertebrae level as the first ventral branch of the abdominal aorta and divides into the left gastric, common hepatic and splenic artery. Many researchers have reported variations of celiac trunk, such as bifurcation, additional branching, combined origin of celiac trunk and superior mesenteric artery (celiacomesenteric trunk), combined origin of celiac trunk, superior and inferior mesenteric artery (celiacobimesenteric trunk) and complete absence [1,2,3,4,5,6,7,8,9,10,11,12,13,14,15,16]. The reported variations in origin and branching pattern of celiac trunk have been found to be valuable for surgical and radiological procedures in the epigastric region [17,18,19,20,21,22,23,24,25,26]. Numerous classification systems have been given for the variations in the origin and branching pattern of the celiac trunk [12,27,28,29], out of which Adachi’s classification is well known, and many arterial patterns of celiac trunk have been reported based on it [29,30,31,32,33,34].

The inferior phrenic artery divides into right and left arterial branches and supplies the diaphragm, esophagus, adrenal gland, inferior vena cava and retroperitoneum. The right and left inferior phrenic arteries normally arise from the aorta or celiac axis independently, or as a common trunk. However, these arteries show several variations depending upon their origin [35,36,37]. Understanding the variations of the inferior phrenic artery are important for surgical and radiological interventions in conditions such as hepatocellular carcinoma, traumatic vascular injuries, hemoptysis and gastroesophageal hemorrhage [35].

The common hepatic artery originates from the celiac trunk and divides into the proper/true hepatic and gastroduodenal artery. Main blood supply to the liver is carried by the hepatic artery; therefore, information about its common and rare variations is essential to avoid injury to the liver and adjacent organs during complex surgical procedures, such as partial hepatectomy, repair of liver rupture and resection of hepatic tumors. Such knowledge may also help during the early postoperative surgeries. In 50–80% of the cases, regular anatomy of the hepatic artery has been reported [7,14,38,39,40,41,42,43,44,45,46,47]. An internationally accepted classification system for anatomical variations of the hepatic artery has been established [7,40,46,48,49,50,51,52]. The classification given by Michels and Hiatts is the most popular for hepatic artery variations [7,40].

Various studies have focused on the vascular supply of the upper gastrointestinal region on the basis of cadaveric dissection. During various pathological conditions, such as tumor, aneurysm, median arcuate ligament syndrome, hepatic resection, splenic rupture in the upper abdominal region and their surgical interventions, it is necessary to know the exact position and variations of the celiac trunk and hepatic artery. These variations can be accurately explored with the help of sophisticated techniques like doppler, ultrasonography, MDCT (multidetector computed tomography) angiography, selective catheter angiography and magnetic resonance angiography [53,54,55]. In this study, variations in the regular anatomy of the celiac trunk and hepatic artery, along with its branches, have been explored by MDCT angiography, which is one of the best techniques to detect even the finest of variations.

The present study gives an account of several variations related to the celiac trunk, hepatic artery branches and inferior phrenic artery through CT angiography in a north Indian population. Five variations related to celiac trunk, three variations related to hepatic artery and one variation related to inferior phrenic artery were observed in the present study. All the variations were co-related with gender and some were found to be more biased towards a specific gender. Comprehension from this work will aid in intricate surgeries related to the upper abdominal region, as well as shine light on variation pattern influence by gender, which may provide several new insights.

## 2. Material and Methods

The present study was performed to determine the origin, course and distribution of the celiac trunk and hepatic artery by the use of 64-row scanner CT angiography in ambulant patients. We analyzed a total number of 110 patients, who were referred to do either triphasic CT (which includes arterial phase) for liver assessment or CT angiography for aortic assessment, during the period from April 2018 to April 2019. Patients were between 18–60 age group belonging to a north Indian population; the study was a side stream of evidence-based patient management, where angiography was performed for clinical symptoms ranging from non-specific abdominal pain to suspected vascular abnormality, such as aortic aneurism. The investigation performed was as part of a clinically relevant indication, depending upon the symptomatology of the patients.

The exclusion criteria applied was the presence of any condition likely to alter the regular vascular anatomy. Ethical clearance was obtained from the Institutional Review Board (Ethical committee registration No. ECR/526/Inst/UP/2014 dated 31 January 2014).

### 2.1. CT Examination

CT angiography was carried out with a General Electric (GE) light speed volumetric computed tomography (VCT) 64- row scanner MDCT angiography machine (GE medical system; Milwauki, WI, USA) and 4.4 ADW version (advantage workstation-GE medical system; Milwauki, WI, USA). The following technical parameters were adopted for each scan-collimation (mm): 64 × 0.6; gantry rotation time: 330 ms; tube voltage: 120 kV; effective tube current: 400 mA; table feed (mm): 46.1; pitch: 1.375:1 55.00 mean scan time: 4.8 s; scan direction: caudocranial direction; reconstruction/increment (mm): 0.6/0.5.

Angiography was performed by injecting 40–60 mL (1 mL/kg body weight) of iodinated contrast medium (omnipaque 350) at the rate of 4.5–5 mL/s, followed by a saline chase of 50 mL at the same rate. Approximate volume of the contrast medium was calculated according to the formula: V = ST × 5 (V = volume enamel, ST = scan time in second).

### 2.2. Image Analysis

In the present study, multiplanar reformation (MPR), maximum intensity projection (MIP) and volume rendering (VR) were used for visualization. In GE VCT 3D workstations, it was possible to display images in three planes simultaneously, to allow for cross referencing and to change the display mode on the fly. MPR images were used for the primary mode of evaluation of central branches. MPR images were particularly used to visualize the celiac trunk. MIPs were created only when a specific projection was selected. VR technique was used for integration of all available information from a volumetric data set with control of the opacity or translucency of selected tissue types.

## 3. Results

### 3.1. Variation in Origin and Branching Pattern of Celiac Trunk

**Figure 1:**
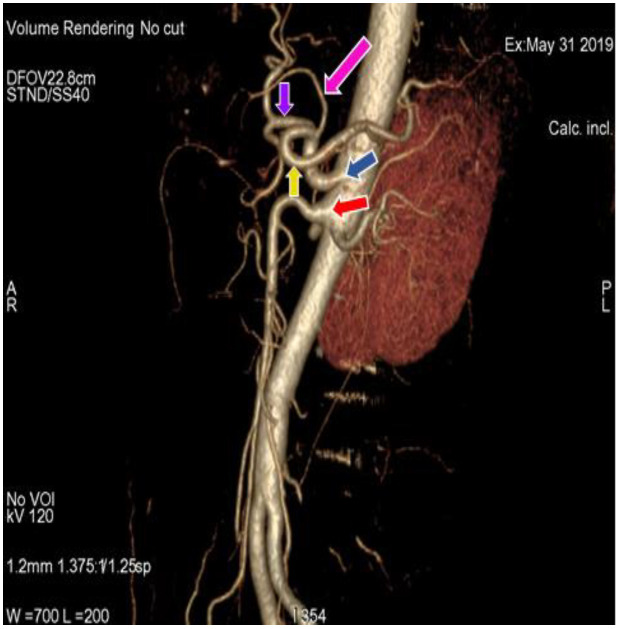
3D-Volume render (3D-VR) image of (64 row scanner CT angiography) abdominal region showing superior mesenteric artery (red arrow) and division of regular celiac trunk (blue arrow) into left gastric artery (pink arrow), splenic artery (yellow arrow) and common hepatic artery (purple arrow).

**Figure 2:**
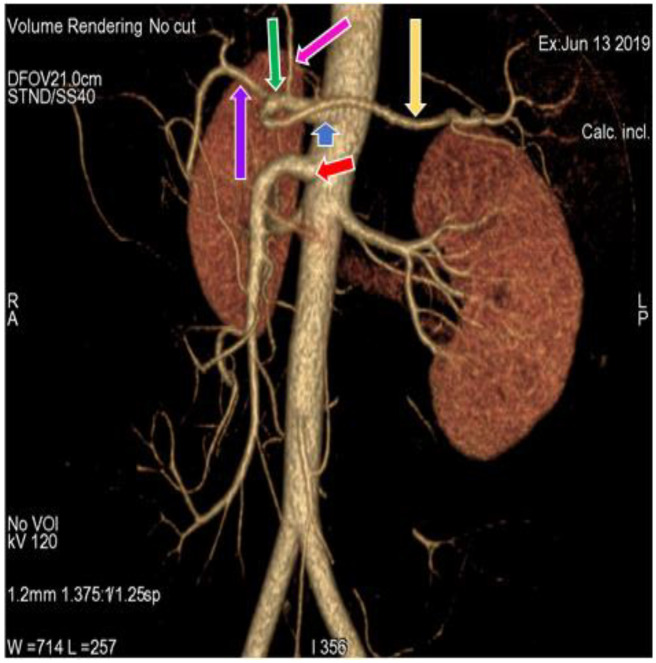
3D-VR image of (64 row scanner CT angiography) abdominal region showing superior mesenteric artery (red arrow) and division of Celiac trunk (blue arrow) into left gastric artery (pink arrow) and hepatosplenic trunk (green arrow). Hepatosplenic trunk further divides into common hepatic artery (purple arrow) and splenic artery (yellow arrow).

**Figure 3:**
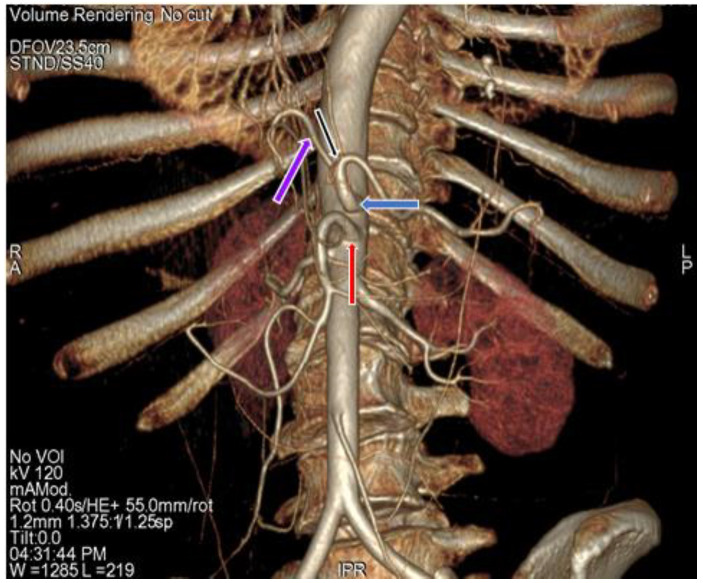
3D-VR image of (64 row scanner CT angiography) abdominal region showing superior mesenteric artery (red arrow) and division of celiac trunk (blue arrow) into gastrosplenic trunk (black arrow) and common hepatic artery (purple arrow).

**Figure 4:**
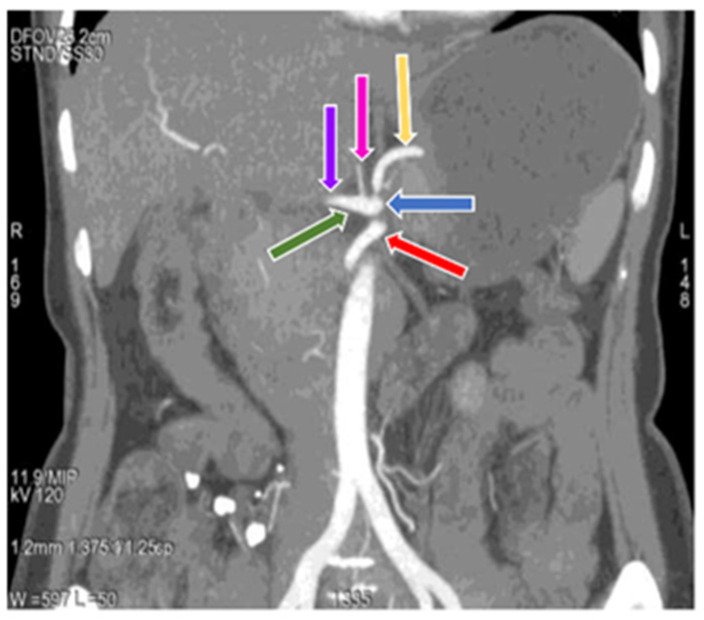
Coronal maximum intensity projection (MIP) image of (64 row scanner CT angiography) abdominal region showing superior mesenteric artery (red arrow) and division of celiac trunk (blue arrow) into gastro-hepatic trunk (dark green arrow) and splenic artery (yellow arrow), gastro-hepatic trunk further divides into common hepatic artery (purple arrow) and left gastric artery (pink arrow). Slice thickness: 1.2 mm.

**Figure 5:**
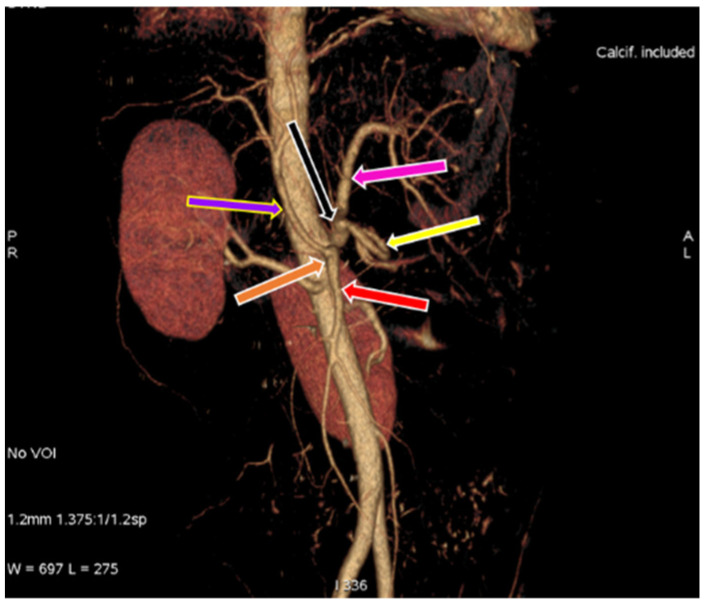
3D-VR image of (64 row scanner CT angiography) abdominal region showing gastrosplenic (black arrow) and hepato-mesenteric trunk (orange arrow), gastrosplenic trunk divides into left gastric artery (pink arrow) and splenic artery (yellow arrow), hepato-mesenteric divides into common hepatic artery (purple arrow) and superior mesenteric artery (red arrow).

**Figure 6:**
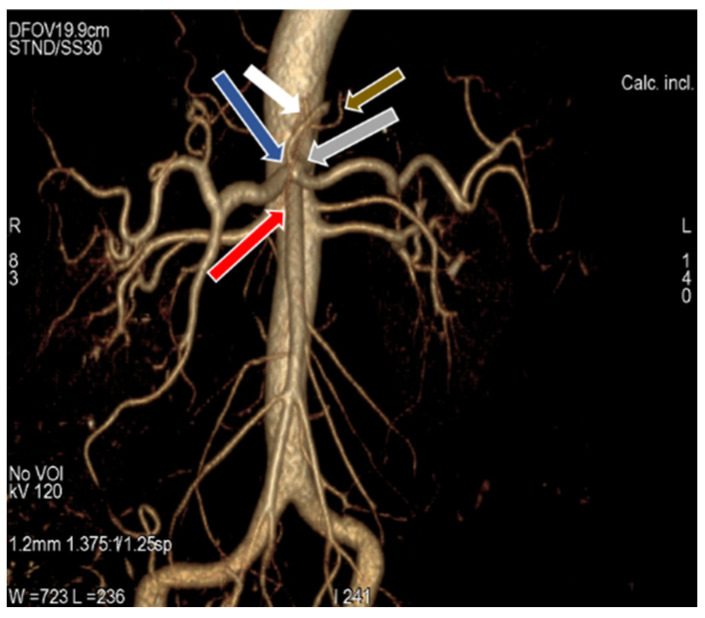
3D-VR image of (64 row scanner CT angiography) abdominal region showing the Celio-mesenteric trunk (grey arrow), which divided into celiac trunk (blue arrow) and superior mesenteric artery (red arrow). Left gastric artery gives rise to right inferior phrenic artery (brown arrow) and left inferior phrenic artery (white arrow).

In the present study, 34 (30.9%) cases (Figure 1) (Table 1) showed a regular origin and branching pattern of the celiac trunk, in which the celiac trunk originated at the level of the T12 thoracic vertebra and divided into the left gastric, splenic and common hepatic artery. In the remaining 76 (69.09%) cases, 5 different patterns of branching of the celiac trunk and superior mesenteric artery were observed. In 66 (60%) cases (Figure 2) (Table 1), the left gastric artery arose just after the origin of the celiac trunk; the celiac trunk continued forward as a hepatosplenic trunk, which further divided into common hepatic and splenic artery. This variation was more common in females (61.4%) than in males (59.1%). In 5 (4.55%) cases (Figure 3) (Table 1) the celiac trunk divided into the gastrosplenic trunk and hepatic artery, and the gastrosplenic trunk further divided into the left gastric artery and the splenic artery. The gastrosplenic trunk was more commonly found in females (6.1%) as compared to males (2.3%). In 2 (1.82%) cases (Figure 4) (Table 1), the celiac trunk originated as hepato-gastric trunk and splenic artery; In addition, the hepato-gastric trunk divided into the common hepatic and splenic artery. The hepato-gastric trunk was more common in males (2.3%) than in females (1.52%). In 2 (1.82%) cases (Figure 5) (Table 1), we observed the gastrosplenic trunk and hepato-mesenteric trunk, in which the gastrosplenic trunk divided into the left gastric and splenic artery; the hepato-mesenteric trunk divided into the common hepatic artery and the superior mesenteric artery. A Gastrosplenic trunk, along with the hepato-mesenteric trunk, was more common in males (2.3%). In 1 (0.91%) case (Figure 6) (Table 1), the celiac trunk and superior mesenteric artery originated from abdominal aorta as a common trunk, known as the celiacomesenteric trunk, and then followed the regular branching pattern. Another interesting variation can be observed in this case, where the right and left inferior phrenic arteries originated from the left gastric artery.

### 3.2. Variation in Origin and Branching Pattern of Hepatic Artery

**Figure 7:**
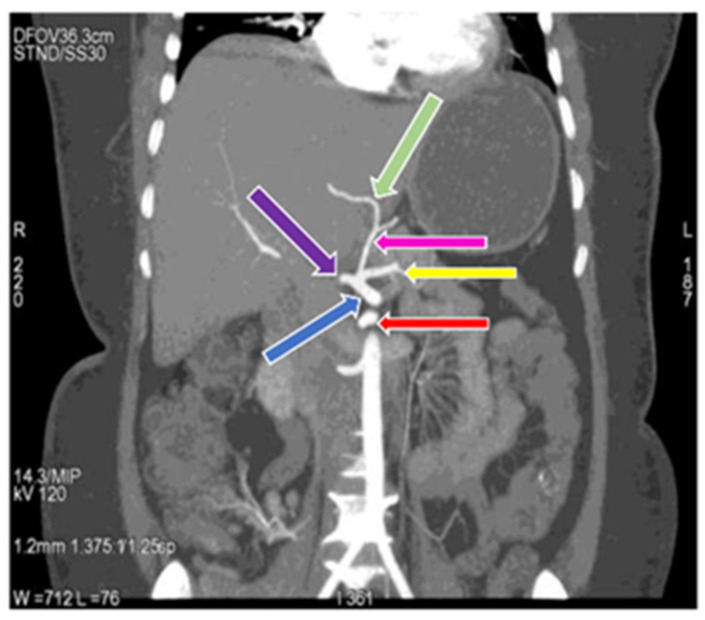
Coronal MIP image of (64 row scanner CT angiography) abdominal region showing the celiac trunk (blue arrow) and superior mesenteric artery (red arrow), the celiac trunk gives rise the left gastric artery (pink arrow), common hepatic artery (purple arrow) and splenic artery (yellow arrow), in which left gastric artery gives rise to left hepatic artery (light green arrow). Slice thickness: 1.2 mm.

**Figure 8:**
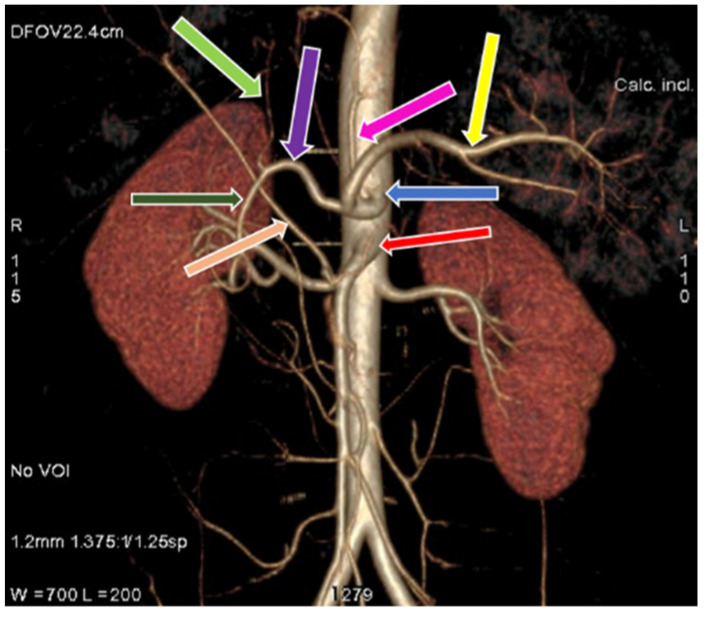
3D-VR image of (64 row scanner CT angiography) abdominal region showing celiac trunk (blue arrow) and superior mesenteric artery (red arrow), common hepatic artery divides into gastroduodenal artery (dark green arrow), left hepatic artery (light green arrow). Replaced right hepatic artery (light pink arrow) originated from superior mesenteric artery. Splenic artery (yellow arrow), left gastric artery (pink arrow), common hepatic artery (purple arrow).

**Figure 9:**
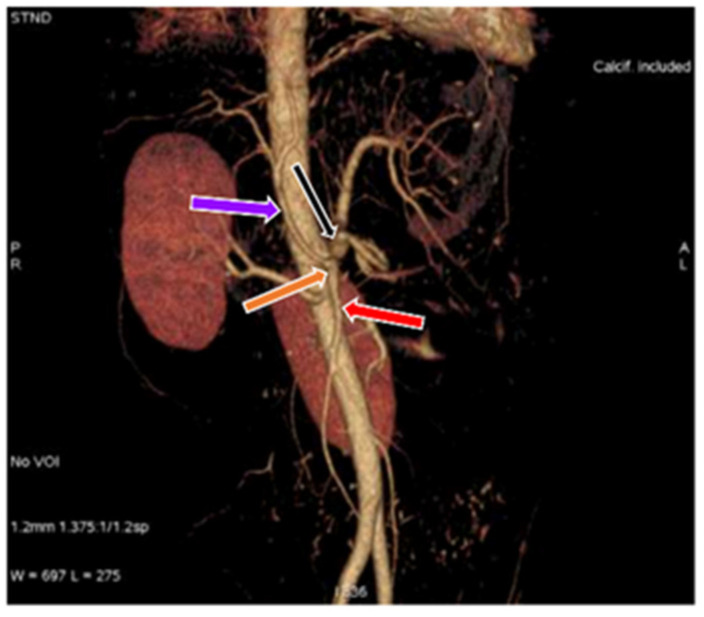
3D-VR image of (64 row scanner CT angiography) abdominal region showing the hepato-mesenteric (orange arrow) and gastrosplenic trunk (black arrow), hepato-mesenteric trunk divides into common hepatic artery (purple arrow) and superior mesenteric artery (red arrow).

The study revealed a regular anatomical pattern of the hepatic artery in 102 (92.73%) cases (Figure 1) (Table 2) (type I variation), in which the common hepatic artery originated from the celiac trunk and gave rise to the true hepatic artery, which further divided into the right and left hepatic artery [7,40]. In two (1.82%) cases (Figure 7) (Table 2) (type II variation), the left hepatic artery was completely replaced and originating from the left gastric artery and the proper hepatic artery continued as the right hepatic artery, seen in only female cases in our study [7,40]. In four (3.64%) cases (Figure 8) (Table 2), the right hepatic artery was completely replaced and originated from the superior mesenteric artery (type III variation); the common hepatic artery gave rise to the left hepatic artery and gastroduodenal artery, with a more common occurrence in males (4.54%) than in females (3.03%) [7,40]. In two (1.82%) cases (Figure 9), (Table 2), we observed the origin of the common hepatic artery from the superior mesenteric artery (type IX Michels and type V Hiatts classification), with a slightly higher occurrence in males (2.3%) than in females (1.52%) [7,40]. Our study did not show any case of Michels classification Type IV, V, VI, VII, VIII, X and Hiatts classification Type IV.

## 4. Discussion

A detailed knowledge of the anatomy and variations of the celiac trunk and the hepatic artery is essential to perform complex surgical procedures related to the upper abdomen [56]. Variants of hepatic and celiac arterial anatomy have been reported by Michels in 1955 [27]. These changes occur at different developmental stages of embryonic life, leading to large variations in abdominal vasculature.

The anatomical variations in the origin of the celiac trunk and its branches are due to embryonic developmental changes in the ventral splanchnic arteries. In the present study, 110 cases have been analyzed by using 64-row scanner CT scan over the period of approximately two years; results revealed five different pattern of celiac trunk (Table 1). In 34 cases (30.9%) out of 110, a regular anatomical pattern of the celiac trunk was observed, which is also known as the true tripod. In comparison with previous studies (43% cases of regular celiac axis anatomy was diagnosed by Brasil et al., 2018; 16.7% cases by Higashi N et al., 2009; 75% cases by Lipshutz. B., 1917; 89% cases by Michels., 1955; 91% cases by Sureka et al., 2013; 86% by Sankar et al., 2011; 85.1% cadaver cases, 89.5% imaging cases, and 95.4%, liver transplantation cases, by Panagouli et al., 2013; 89.1% cases by Song et al., 2010; 89.8% cases by Chen et al., 2009; 90% cases by Neto et al., 2015), the present study reported a lower percentage of a regular pattern of the celiac trunk, which may depend upon the region of the population and sample size taken [12,15,27,48,50,57,58,59,60,61]. In the present study, variations of the celiac trunk were diagnosed in 69.1% cases, which was higher in contrast to 10.6%, reported by Panagouli et al., 2013, 5.5% reported by Sureka et al., 2013, 14% reported by Sankar et al., 2011, 9.6% reported by Song et al., 2010, and 10.2% reported by Chen et al., 2009 earlier [48,50,58,59,60]. In the present study, 66 cases (60%) out of 110 cases showed the hepatosplenic trunk and left gastric artery as the first branch of the celiac trunk and superior mesenteric artery from the abdominal aorta. This variation was observed more in male patients as compared to females; however, no significant difference could be found. In the literature, many cases of hepatosplenic trunk have been previously reported: Sureka et al., 2013 in 2.3% cases, Michels., 1955 in 4% cases, Song et al., 2010 in 4.42% cases, and Neto et al., 2015 in 4.8% of cases [27,50,60,61]. In our second study, common variation was present in five (4.55%) cases, in which celiac trunk was divided in gastrosplenic trunk and common hepatic artery as first branch of celiac trunk. This pattern was significantly higher in female patients as compared to males. In the previous literature, Eaton., 1917, Michels., 1955 and Ugural et al., 2010 reported this variation as the most common variation [10,27,62]. Another type of celiac trunk variation was present in two cases in the present study (1.82%), in which celiac trunk is divided into hepato-gastric trunk and splenic artery. This variation was non-significantly higher in male subjects. This is a rare type of variation and also called as false tripod. Another type of celiac trunk variation is found where the true celiac trunk is absent and there is a presence of the gastrosplenic and the hepato-mesenteric trunk. Very few cases were found in the literature regarding this variation. In our study, this type of variation was found to be non-significantly higher in males. A very rare variation in which celiac trunk unites with the superior mesenteric artery at their origin from abdominal aorta to form a celiacomesenteric trunk, corresponds to type IV variation [63]. In the present study, one case (0.91%) of the celiacomesenteric trunk was diagnosed in female patients. In the literature, very few cases of the celiacomesenteric trunk have been reported [64]. Celiacomesenteric trunk has various surgical and medical implications. Since, in the celiacomesenteric trunk, a single trunk arises from abdominal aorta; hence, it lacks the protective benefits of separate origin vessels and their anastomosis channels. This increases the risk of vascular compromise of abdominal viscera during major abdominal surgeries. Although presence of celiacomesenteric trunk was observed in female case, no definitive correlation could be drawn between the presence of celiacomesenteric trunk and gender due to limited sample size which, is in accordance with the previous studies [65,66]. There are various studies describing the relationship between the inferior phrenic artery and the celiac trunk. The inferior phrenic artery originates not only from aorta, celiac trunk and its branches but also from many other different arteries. Basile et al. described 13 different type of inferior phrenic artery variations through MDCT and Loukas et al. classified the origin of inferior phrenic artery in five groups on the basis of cadaveric dissection. The origin of inferior phrenic artery is important in various clinical pathological conditions, most commonly in hepatocellular carcinoma and gastroesophageal bleeding management [67,68].

Hepatic neoplasm receives blood supply from the hepatic arteries and some extrahepatic collateral arteries, such as inferior phrenic artery [35]. In our study, right and left inferior phrenic artery originated from the left gastric artery, as observed in 0.91% of cases. According to Piao et al., the inferior phrenic artery originates from the aorta in 61.6% cases, from the celiac trunk in 28.2% cases and the remaining 10.19% arises from either renal artery, left gastric or middle adrenal arteries. In another study, Pulakunta et al. reported that the inferior phrenic artery originated from the celiac trunk in 6.25% cases, left gastric artery in 3.125% cases, right renal artery in 3.125% cases and 87.5% cases had the usual aortic origin [69,70,71]. Many cases of hepatic artery and its variations have been reported in the literature. In the present study, Michels and Hiatts’ classification system were used to study the anatomical variation of the hepatic artery [7,40]. Zagyapan et al., 2014 observed 37.5% cases of hepatic artery variations by digital angiography [72]. 77.2% cases of regular hepatic arterial anatomy, and 22.8% cases of variant hepatic artery anatomy were reported in 1200 cases by Kobayashi et al., 2014 [56]. Michels type I classification was most common with regular anatomy of the hepatic artery in the present study, 102 cases (92.72%) (Table 2) had regular hepatic anatomy, which was non-significantly different in both males and females and in agreement with the already present literature. Gumus et al., 2013, Sureka et al., 2013, Sebben et al., 2013, Chen et al., 2009 and Freitas et al., 2001 reported Michels type I pattern in 66.8%, 55%, 73%, 51% and 76.82% cases, respectively [15,40,48,49,50,73]. In our study, eight cases (7.28%) showed variation in hepatic artery anatomy. According to Michels classification system, the most frequent hepatic artery variation is the type III present in 6–15% of cases [40]. In the present study this was also the most frequent variation, present in 3.64% of cases and was non-significantly higher in males (Table 2). The second most common variation is type II, reported in literature between 2.5–10%, in which left hepatic artery is completely replaced and originated from left gastric artery. Knowledge of this variation is utmost important during left lobe hepatectomy and in the present study this variation was found only in female cases. The types VII, VIII, IX and X are rarely described in the literature [3,39,62]. These cases were also not observed in present study. According to Hiatt classification, type III is most common and present in 10.6% of cases and in the present study this variation was diagnosed in 3.64% cases and type II is second most common and present in 9.7% cases; In present study this variation was diagnosed in 1.82% cases. Both these variations were found to be non-significantly higher in males. According to both Michels and Hiatt classification, in type III variation right hepatic artery is completely replaced and (Figure 8) originates from superior mesenteric artery while common hepatic artery gives rise to the left gastric and gastroduodenal artery [3,38,74]. Gastroduodenal artery follows downward course and supplies the head of pancreas and part of greater curvature of stomach.

In conclusion to deal with various pathological and emergency surgical conditions of the upper abdominal region, it is necessary to know the precise anatomical position of the celiac trunk, hepatic artery and inferior phrenic artery. Celiac trunk and hepatic artery variations are vital in hepato-biliary surgeries, pancreatic surgeries and hepatic arteriography to avoid injuries to these vital organs. Knowledge of inferior phrenic artery variations is important for laparoscopic surgeries, diagnostic-interventional radiology, gastroesophageal bleeding management and transplantation. With the advancement of technology, MDCT angiography has become the gold standard in detecting any minute vessels abnormalities and their exact position thus increasing the surgical precision many folds. Gender-based variation has been explored less due to non-significant differences in the variations. However, in the present study some variations were found to be more biased towards a specific gender while others were found to be non-significant. The present study provides an overview of variations found in the celiac trunk and hepatic artery in north Indian population, along with gender-based variations, which will be helpful in various surgical procedures of the upper abdominal region.

## Figures and Tables

**Table 1:** Variations in origin and branching pattern of celiac trunk and superior mesenteric artery.

Anatomical Pattern of Celiac Trunk	Female Cases (66)	Male Cases (44)	Total Number of Cases
Regular celiac trunk	20 (30.3%)	14 (31.82%)	34 (30.9%)
Hepatosplenic trunk and Left gastric artery	39 (59.1%)	27 (61.4%)	66 (60%)
Gastrosplenic trunk and Common hepatic artery	4 (6.1%)	1 (2.3%)	5 (4.55%)
Heapto-gastric trunk and splenic artery	1 (1.52%)	1 (2.3%)	2 (1.82%)
Gastrosplenic and Hepato-mesentric trunk	1 (1.52%)	1 (2.3%)	2 (1.82%)
Celiacomesentric trunk	1 (1.52%)	0 (0%)	1 (0.91%)

**Table 2:** Variations in origin and branching pattern of hepatic artery according to Michels and Hiatts classification.

Anatomical Variation of Hepatic Artery	Total Number of Cases (110)	Total Female Cases (66)	Total Male Cases (44)	Michels Classification	Hiatts Classification
Regular anatomy	102 (92.72%)	61 (92.43%)	41 (93.18%)	Type I	Type I
LHA branch LGA	2 (1.82%)	2 (3.03%)	0 (0%)	Type II	Type II
RHA branch SMA	4 (3.64%)	2 (3.03%)	2 (4.54%)	Type III	Type III
Type I and II association	0	0	0	Type IV	Type IV
LHA accessory LGA	0	0	0	Type V	Type II
RHA accessory SMA	0	0	0	Type VI	Type III
LHA accessory LGA + RHA accessory SMA	0	0	0	Type VII	Type IV
LHA accessory LGA + RHA branch SMA	0	0	0	Type VIII	Type IV
CHA branch SMA	2 (1.82%)	1 (1.52%)	1 (2.3%)	Type IX	Type V
RHA and LHA branch LGA	0	0	0	Type X	-
CHA aorta branch	0	0	0	-	Type VI
